# Charm and beauty quark masses in the MMHT2014 global PDF analysis

**DOI:** 10.1140/epjc/s10052-015-3843-5

**Published:** 2016-01-06

**Authors:** L. A. Harland-Lang, A. D. Martin, P. Motylinski, R. S. Thorne

**Affiliations:** Department of Physics and Astronomy, University College London, London, WC1E 6BT UK; Institute for Particle Physics Phenomenology, Durham University, Durham, DH1 3LE UK

## Abstract

We investigate the variation in the MMHT2014 PDFs when we allow the heavy-quark masses $m_c$ and $m_b$ to vary away from their default values. We make PDF sets available in steps of $\Delta m_c =0.05~\mathrm{GeV}$ and $\Delta m_b =0.25~\mathrm{GeV}$, and present the variation in the PDFs and in the predictions. We examine the comparison to the HERA data on charm and beauty structure functions and note that in each case the heavy-quark data, and the inclusive data, have a slight preference for lower masses than our default values. We provide PDF sets with three and four active quark flavours, as well as the standard value of five flavours. We use the pole mass definition of the quark masses, as in the default MMHT2014 analysis, but briefly comment on the $\overline{\mathrm{MS}}$ definition.

## Introduction

Over the past few years there has been a significant improvement both in the precision and in the variety of the data for deep-inelastic and related hard-scattering processes. Since the MSTW2008 analysis [1] we have seen the appearance of the HERA *combined* H1 and ZEUS data on the total [2] and also on the charm structure functions [3], together with a variety of new hadron-collider data sets from the LHC, and in the form of updated Tevatron data (for full references see [4]). Additionally, the procedures used in the global PDF analyses of data have been improved, allowing the parton distributions of the proton to be determined with more precision and with more confidence. This allows us to improve predictions for Standard Model signals and to model Standard Model backgrounds to possible experimental signals of New Physics more accurately. One area that now needs careful attention, at the present level of accuracy, is the treatment of the masses of the charm and beauty quarks, $m_c$ and $m_b$, in the global analyses. Here we extend the recent MMHT2014 global PDF analysis [4] to study the dependence of the PDFs, and the quality of the comparison to data, under variations of these masses away from their default values of $m_c=1.4~\mathrm{GeV}$ and $m_b=4.75~\mathrm{GeV}$, as well as the resulting predictions for processes at the LHC. We make available central PDF sets for a variety of masses, namely $m_c=$ 1.15–1.55 GeV in steps of $0.05~\mathrm{GeV}$ and $m_b=$ 4.25–5.25 GeV in steps of $0.25~\mathrm{GeV}$. We also make available the standard MMHT2014 PDFs, and the sets with varied masses in the three and four flavour number schemes.

**Fig. 1:**
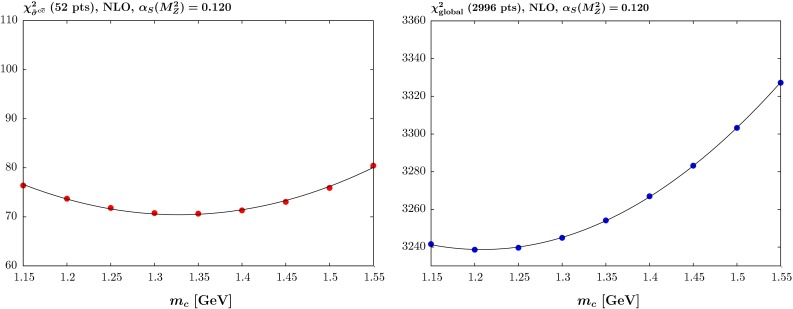
The quality of the fit versus the quark mass $m_c$ at NLO with $\alpha _S(M_Z^2)=0.120$ for (*left*) the reduced cross section for charm production $\tilde{\sigma }^{c\bar{c}}$ for the combined H1 and ZEUS data and (*right*) the full global fit

## Dependence on the heavy-quark masses

### Choice of range of heavy-quark masses

In the study of heavy-quark masses that accompanied the MSTW2008 PDFs [5] we varied the charm and beauty quark masses, defined in the pole mass scheme, from $m_c=1.05$ to 1.75 $\mathrm{GeV}$ and $m_b=4$ to 5.5 $\mathrm{GeV}$. This was a very generous range of masses, and it was not clear that there was a demand for PDFs at the extreme limits. Hence, this time we are a little more restrictive, and study the effects of varying $m_c$ from 1.15 to $1.55~\mathrm{GeV}$, in steps of $0.05~\mathrm{GeV}$, and of varying $m_b$ from 4.25 to $5.25~\mathrm{GeV}$ in steps of $0.25~\mathrm{GeV}$. Part of the reason for this is that the values are constrained by the comparison to data, though for both charm and beauty the preferred values are at the lower end of the range, as we will show. However, there is also the constraint from other determinations of the quark masses. These are generally quoted in the $\overline{\mathrm{MS}}$ scheme, and in [6] are given as $m_c(m_c)=(1.275 \pm 0.025)~\mathrm{GeV}$ and $m_b(m_b)=(4.18\pm 0.03)~\mathrm{GeV}$. The transformation to the pole mass definition is not well defined due to the diverging series, i.e. there is a renormalon ambiguity of $\sim$0.1–0.2 $\mathrm{GeV}$. The series is less convergent for the charm quark, due to the lower scale in the coupling, but the renormalon ambiguity cancels in difference between the charm and beauty masses. Indeed, we obtain $m_b^\mathrm{pole}-m_c^\mathrm{pole}=3.4~\mathrm{GeV}$ with a very small uncertainty [7, 8]. Using the perturbative expression for the conversion of the beauty mass, and the relationship between the beauty and charm mass, as shown in [5], we obtain

1 $$\begin{aligned} m_c^\mathrm{pole}=1.5 \pm 0.2 ~{\mathrm{GeV}} \quad \mathrm{and} \quad m_b^\mathrm{pole}=4.9\pm 0.2~\mathrm{GeV}. \end{aligned}$$

This disfavours $m_c\le$ 1.2–1.3 $\mathrm{GeV}$ and $m_b\le$ 4.6–4.7 $\mathrm{GeV}$. As the fit quality prefers values in this region, or lower, we allow some values a little lower than this. In the upper direction the fit quality clearly deteriorates, so our upper values are not far beyond the central values quoted above. There is some indication from PDF fits for a slightly lower $m^\mathrm{pole}$ than that suggested by the use of the perturbative series out to the order at which it starts to show lack of convergence. We now consider the variation with $m_c$ and $m_b$ in more detail.

### Dependence on $m_c$

We repeat the global analysis in [4] for values of $m_c=$ 1.15–1.55 $\mathrm{GeV}$ in steps of $0.05~\mathrm{GeV}$. As in [4] we use the “optimal” version [9] of the TR’ general-mass variable-flavour-number scheme GM-VFNS [10]. This is smoother near the transition point, which we define to be at $Q^2=\mu ^2 =m_c^2$, than the original version, so has a slight tendency to prefer lower masses—the older version growing a little more quickly at low scales, which could be countered by increasing the mass. We also assume all heavy flavour is generated by evolution from the gluon and light quarks, i.e. there is no intrinsic heavy flavour. We perform the analysis with $\alpha _S(M_Z^2)$ left as a free parameter in the fit at both NLO and NNLO, but also use our fixed default values of the coupling of $\alpha _S(M_Z^2)=0.118$ and 0.120 at NLO and $\alpha _S(M_Z^2)=0.118$ at NNLO. Unlike the MSTW2008 study [5] we will concentrate on the results and PDFs with fixed coupling, as the standard MMHT PDFs were made available at these values.

**Fig. 2:**
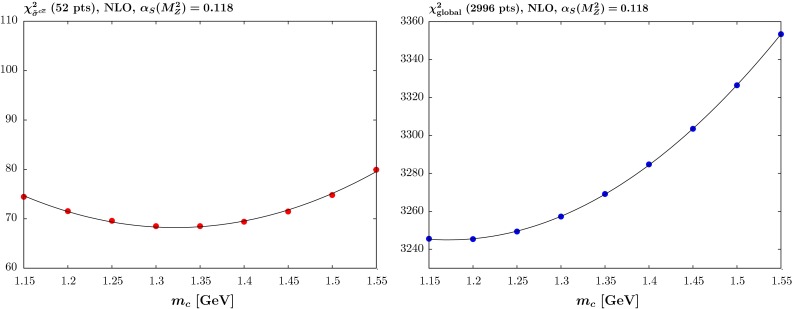
The quality of the fit versus the quark mass $m_c$ at NLO with $\alpha _S(M_Z^2)=0.118$ for (*left*) the reduced cross section for charm production $\tilde{\sigma }^{c\bar{c}}$ for the combined H1 and ZEUS data and (*right*) the full global fit

We present results in terms of the $\chi ^2$ for the total set of data in the global fit and for just the data on the reduced cross section, $\tilde{\sigma }^{c\bar{c}}$, for open charm production at HERA [3]. This is shown at NLO with $\alpha _S(M_Z^2)=0.120$ in Fig. 1. The variation in the quality of the fit to the HERA *combined* charm cross section data is relatively slight, less than the variation in the fit to the *separate* H1 and ZEUS data used in [5]. This is presumably due to the use of the full information now available on correlated systematics, which allows movement of the data relative to the theory with only a moderate penalty in $\chi ^2$. The lower variation is also likely due in part to the improved flavour scheme. Despite the fairly small variation in $\chi ^2$ the charm data clearly prefer a value close to $m_c=1.35~\mathrm{GeV}$, near our default value of $m_c=1.4~\mathrm{GeV}$. However, there is more variation in the fit quality to the global data set, with a clear preference for values close to $m_c=1.2~\mathrm{GeV}$. The deterioration is clearly such as to make values of $m_c>1.5~\mathrm{GeV}$ strongly disfavoured. The main constraint comes from the inclusive HERA cross section data, but there is also some preference for a low value of the mass from NMC structure function data, where the data for $x\sim 0.01$ and $Q^2\sim 4~\mathrm{GeV}^2$ is sensitive to the turn-on of the charm contribution to the structure function. Overall, there is some element of tension between the preferred value for the global fit and the fit to charm data. We do not attempt to make a rigorous determination of the best value of the mass or its uncertainty, as provided in [11] for example, as we believe there are more precise and better controlled methods for this. However, a rough indication of the uncertainty could be obtained from the $\chi ^2$ profiles by treating $m_c$ in the same manner as the standard PDF eigenvectors and applying the dynamic tolerance procedure. In this case the appropriate tolerance, obtained by assuming the charm cross section data is the dominant constraint, would be of the order $T=\sqrt{\Delta \chi ^2}\approx 2.5$.^1^

**Table 1:** The quality of the fit versus the quark mass $m_c$ at NLO with $\alpha _S(M_Z^2)$ left as a free parameter

$m_c$ (GeV)	$\chi ^2_\mathrm{global}$	$\chi ^2_{\tilde{\sigma }^{c\bar{c}}}$	$\alpha _s(M_Z^2)$
	2996 pts	52 pts	
1.15	3239	75	0.1190
1.2	3237	73	0.1192
1.25	3239	71	0.1194
1.3	3245	70	0.1195
1.35	3254	70	0.1196
1.4	3268	71	0.1198
1.45	3283	73	0.1200
1.5	3303	76	0.1201
1.55	3327	81	0.1202

**Fig. 3:**
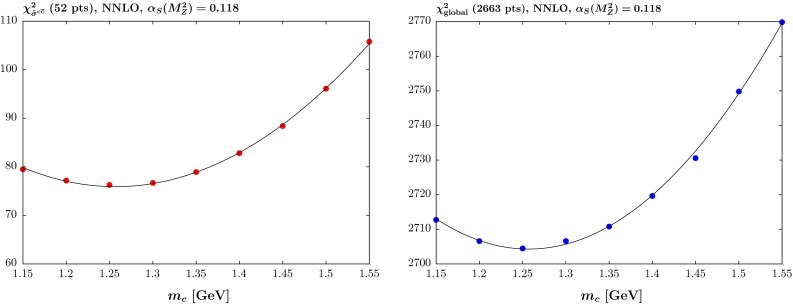
The quality of the fit versus the quark mass $m_c$ at NNLO with $\alpha _S(M_Z^2)=0.118$ for (*left*) the reduced cross section for charm production $\tilde{\sigma }^{c\bar{c}}$ for the combined H1 and ZEUS data and (*right*) the full global fit

The analogous results for $\alpha _S(M_Z^2)=0.118$ and $\alpha _S(M_Z^2)$ left free are shown in Fig. 2 and Table 1, respectively, where in the latter case the corresponding $\alpha _s(M_Z^2)$ values are shown as well. For $\alpha _S(M_Z^2)=0.118$ the picture is much the same as for $\alpha _S(M_Z^2)=0.120$ except that the fit to charm data is marginally better, while the global fit is a little worse, though more-so for higher masses. The results with free $\alpha _S(M_Z^2)$ are consistent with this, with the preferred value of $\alpha _S(M_Z^2)$ falling slightly with lower values of $m_c$. However, the values of $m_c$ preferred by charm data and the full data sets are much the same as for fixed coupling—the values of the $\chi ^2$ just being a little lower in general.

The results of the same analysis at NNLO are shown for $\alpha _S(M_Z^2)=0.118$ and $\alpha _S(M_Z^2)$ left free in Fig. 3 and Table 2, respectively, where again in the latter case the corresponding $\alpha _s(M_Z^2)$ values are shown. Broadly speaking, the results are similar to those at NLO, but with lower values of $m_c$ preferred and where the $\chi ^2$ variation is greater for the inclusive data than for the charm cross section data. However, in this case there is essentially no tension at all between the inclusive and charm data, with both $\chi ^2$ values minimising very near to $m_c=1.25~\mathrm{GeV}$—this lower preferred value for the charm data meaning that the fit quality at $m_c=1.55~\mathrm{GeV}$ has deteriorated more than at NLO. The picture is exactly the same for fixed and free strong coupling, with the values of $\chi ^2$ simply being a little lower when $\alpha _S(M_Z^2)$ is left free, since the best fit value of the coupling is a little below 0.118, particularly for low $m_c$.

**Table 2:** The quality of the fit versus the quark mass $m_c$ at NNLO with $\alpha _S(M_Z^2)$ left free

$m_c$ (GeV)	$\chi ^2_\mathrm{global}$	$\chi ^2_{\tilde{\sigma }^{c\bar{c}}}$	$\alpha _s(M_Z^2)$
	2663 pts	52 pts	
1.15	2703	78	0.1164
1.2	2699	76	0.1166
1.25	2698	75	0.1167
1.3	2701	76	0.1169
1.35	2707	78	0.1171
1.4	2717	82	0.1172
1.45	2729	88	0.1173
1.5	2749	96	0.1173
1.55	2769	105	0.1175

### Dependence on $m_b$

We repeat essentially the same procedure for varying values of $m_b$ in the range 4.25–5.25 $\mathrm{GeV}$ in steps of $0.25\,\mathrm{GeV}$. However, this time there were no data on the beauty contribution to the cross section included in the standard global fit [4]. In the previous heavy-quark analysis [5] we compared to beauty cross section data from H1 [12]. This placed a weak constraint on the value of $m_b$ but had negligible constraint on the PDFs for fixed $m_b$. Hence, we did not include these data in the updated global fit [4]. There are now also data of comparable precision from ZEUS [13], and we will include both these data sets in future global fits. In this article we study the quality of the comparison to these data to predictions obtained using the MMHT PDFs with different values of $m_b$. The data themselves are not included in the fit, i.e. we use predictions from the PDFs, as they still provide negligible direct constraint.

**Fig. 4:**
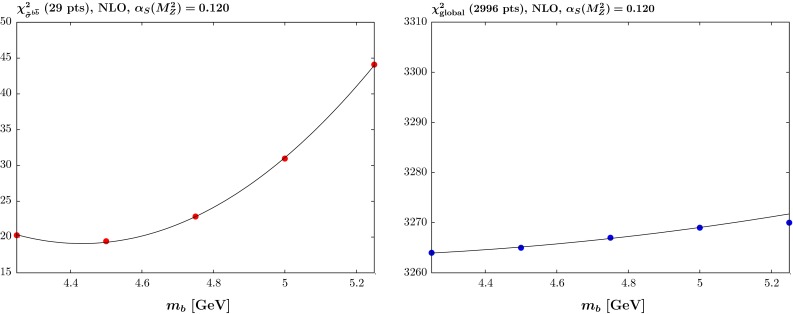
The quality of the fit versus the quark mass $m_b$ at NLO with $\alpha _S(M_Z^2)=0.120$ for (*left*) the reduced cross section for beauty production $\tilde{\sigma }^{b\bar{b}}$ for the H1 and ZEUS data and (*right*) the global fit, not including the beauty data

**Fig. 5:**
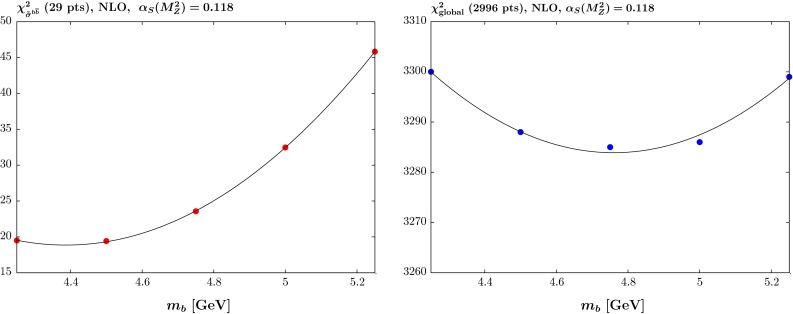
The quality of the fit versus the quark mass $m_b$ at NLO with $\alpha _S(M_Z^2)=0.118$ for (*left*) the reduced cross section for beauty production $\tilde{\sigma }^{b\bar{b}}$ for the H1 and ZEUS data and (*right*) the global fit, not including the beauty data

**Fig. 6:**
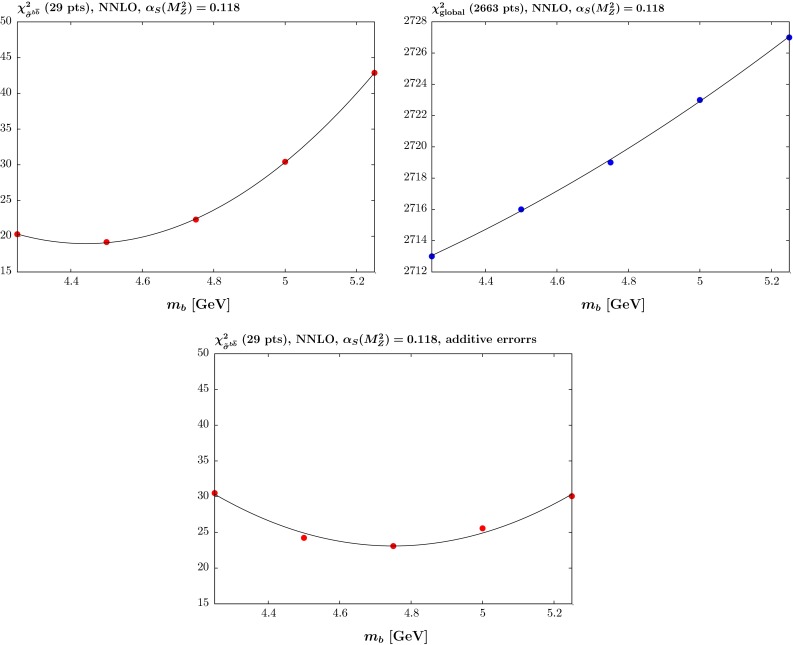
The quality of the fit versus the quark mass $m_b$ at NNLO with $\alpha _S(M_Z^2)=0.118$ for (*left*) the reduced cross section for beauty production $\tilde{\sigma }^{b\bar{b}}$ for the H1 and ZEUS data and (*right*) the global fit, not including the beauty data. Recall that in the MMHT analysis the experimental errors are treated multiplicatively. The *lower plot* shows the $\chi ^2$ profile if the errors in the HERA beauty data were to be treated additively

The results for the NLO PDFs with $\alpha _S(M_Z^2)=0.120$ and 0.118 are shown in Figs. 4 and  5, respectively. The picture for the data in the global fit (not including the $\tilde{\sigma }^{b\bar{b}}$ data) is slightly different in the two cases: for $\alpha _S(M_Z^2)=0.120$ there is a fairly weak tendency to prefer lower values of $m_b$, similar to the results in [5], but for $\alpha _S(M_Z^2)=0.118$ the global fit prefers a value of between 4.5 and $5.0~\mathrm{GeV}$. For the predictions for the beauty cross section data, however, the picture is similar in the two cases, and low values of $m_b \sim$ 4.4–4.5 $\mathrm{GeV}$ are preferred.

The results for the NNLO fit with $\alpha _S(M_Z^2)=0.118$ are shown in Fig. 6. As can be seen the global fit is fairly weakly dependent on $m_b$, though more than for $\alpha _S(M_Z^2)=0.120$ at NLO, and prefers a value lower than $m_b=4.25~\mathrm{GeV}$. As in the NLO case the $\chi ^2$ for the prediction for $\tilde{\sigma }^{b\bar{b}}$ is better for lower values of $m_b$. The slightly larger variations in the quality of the global fit with varying $m_b$ compared to [5] is perhaps due to the greater precision of the inclusive HERA cross section data used in this analysis, and to the fact that the CMS double-differential Drell–Yan data [14] has some sensitivity to the value of $m_b$ due to the induced variation in sea quark flavour composition for low scales. The previous analysis preferred a value of $m_b \sim 4.75~\mathrm{GeV}$ for the comparison to the H1 beauty data. However, the definition of the general-mass variable-number scheme has improved since this previous analysis, being smoother near to the transition point $Q^2=m_b^2$, and including an improvement to the approximation for the $\mathcal{O}(\alpha _S^3)$ contribution at low $Q^2$ at NNLO, so some changes are not surprising. Another important difference is in the treatment of the correlated experimental errors, which we now take as being multiplicative. The result within exactly the same framework, but with the experimental errors on the HERA beauty data instead treated as additive is also shown in Fig. 6 and a higher value of $m_b \sim 4.75~\mathrm{GeV}$ is clearly preferred. Similar results are seen in the NLO fits.

**Fig. 7:**
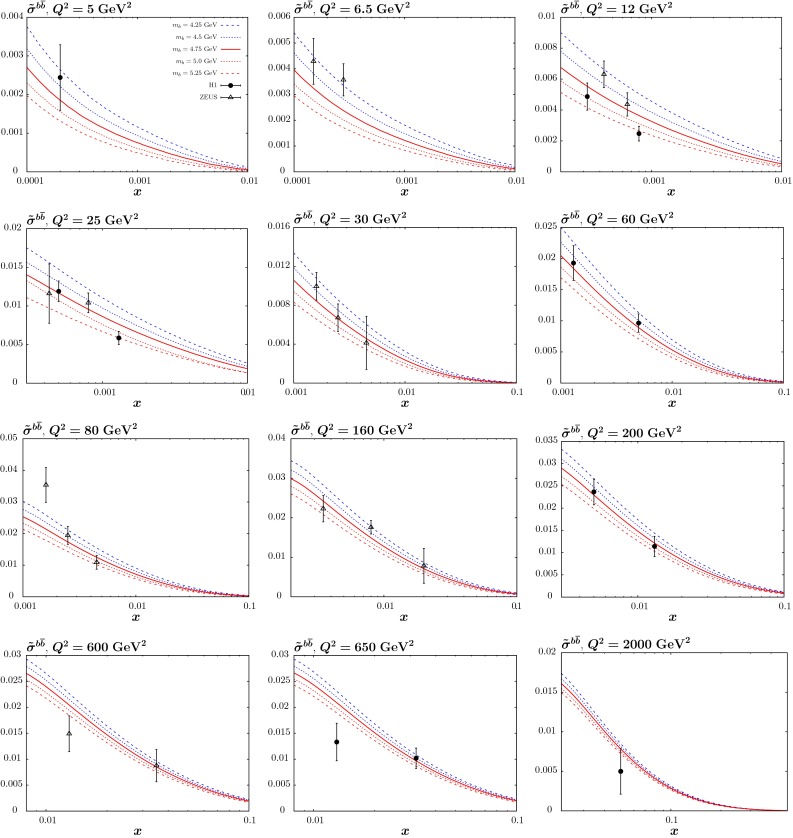
The (unshifted) HERA $\tilde{\sigma }(b{\bar{b}})$ data versus *x* at 12 different values of $Q^2$, namely $Q^2=5,6.5,\ldots , 2000$ GeV$^2$; the H1 [12] and ZEUS [13] data are shown as *solid circular* and *clear triangular points*, respectively. The *curves* are the NNLO predictions for five different values of $m_b$, namely, in descending order, $m_b=4.25,4.5,4.75,5,5.25$ GeV

In Fig. 7 the comparison to the (unshifted) HERA beauty data for different values of $m_b$ at NNLO is shown. At low $Q^2$ and for ZEUS data in particular, the curves for lower $m_b$ are clearly a better fit to unshifted data. However, the low-$m_b^2$ predictions do significantly overshoot some of the unshifted data points. These predictions will work better with the multiplicative definition of uncertainties as the size of the correlated uncertainties then scales with the prediction, not the data point (as would be the case in the additive definition), or equivalently, if data are normalised up to match theory, then so is the uncorrelated uncertainty.

### Changes in the PDFs

**Fig. 8:**
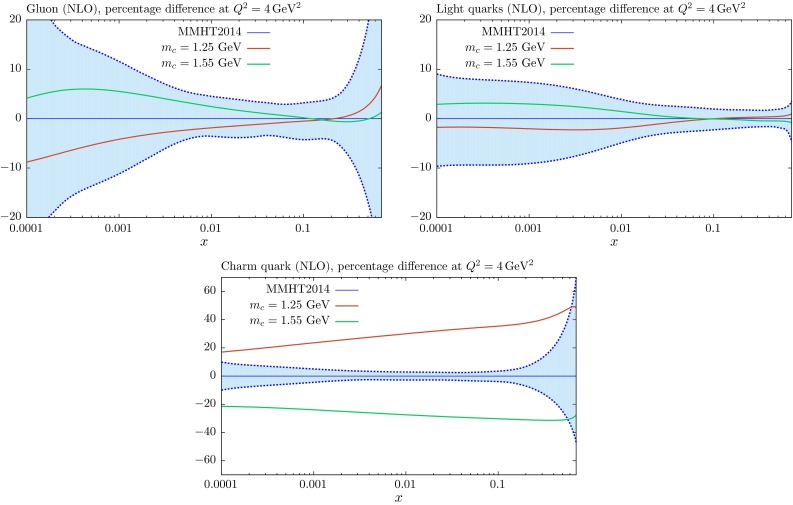
The $m_c$ dependence of the gluon, light-quark singlet and charm distributions at NLO for $Q^2=4~\mathrm{GeV}^2$, compared to the standard MMHT2014 distributions with $m_c=1.4~\mathrm{GeV}$ and $m_b=4.75~\mathrm{GeV}$

**Fig. 9:**
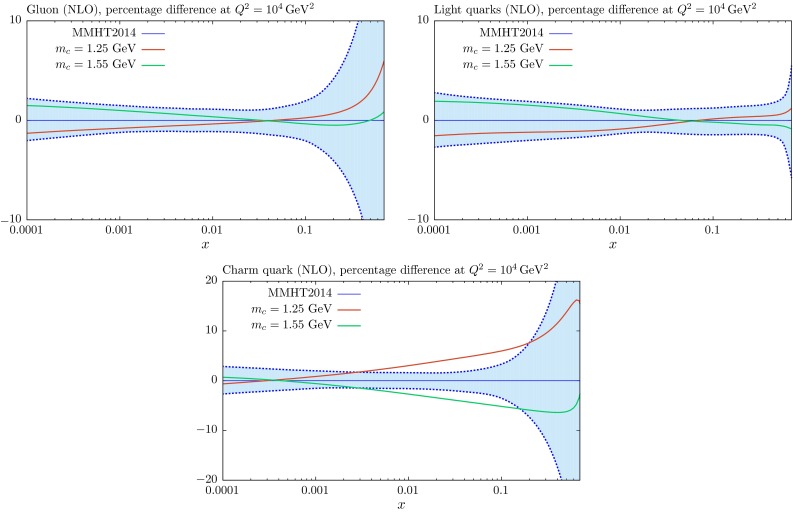
The $m_c$ dependence of the gluon, light-quark singlet and charm distributions at NLO for $Q^2=10^4~\mathrm{GeV}^2$, compared to the standard MMHT2014 distributions with $m_c=1.4~\mathrm{GeV}$ and $m_b=4.75~\mathrm{GeV}$

We show how the NLO PDFs for $m_c=1.25$ and $1.55~\mathrm{GeV}$ compare to the central PDFs in Figs. 8 and 9. Results are very similar at NNLO, though more complicated to interpret for the charm distribution at low $Q^2$ due to the non-zero transition matrix element at $Q^2=m_c^2$ in this case. We see at $Q^2=4$ GeV$^2$ (that is, close to the transition point $Q^2=m_c^2$) that the change in the gluon is well within its uncertainty band, though there is a slight increase at smaller *x* with higher $m_c$ (and *vice versa*) such that extra gluon quickens the evolution of the structure function which is suppressed by larger mass. Similarly the light quark singlet distribution increases slightly near the transition point for larger $m_c$ to make up for the smaller charm contribution to structure functions, and this is maintained, helped by the increased gluon, at larger scales. In both cases, however, the changes are within uncertainties for these moderate variations in $m_c$. The charm distribution increases at low $Q^2$ for decreasing $m_c$, and *vice versa*, simply due to increased evolution length $\ln (Q^2/m_c^2)$. As mentioned before we have identified the transition point at which heavy flavour evolution begins with the quark mass. This has the advantage that the boundary condition for evolution is zero up to NLO (with our further assumption that there is no intrinsic charm), though there is a finite $\mathcal{O}(\alpha _S^2)$ boundary condition at NNLO in the GM-VFNS, available in [15]. In principle the results on the charm distribution at relatively low scales, such as that in Fig. 8 are sensitive to these definitions at finite order, though as the order in QCD increases the correction for changes due to different choices of the transition point arising from the corresponding changes in the boundary conditions become smaller and smaller, ambiguities always being of higher order than the calculation. At scales typical of most of LHC physics, however, the relative change in evolution length for the charm distribution is much reduced, as are the residual effects of choices relating to the choice of the transition point and intrinsic charm. At these scales the change in the charm distribution is of the same general size as the PDF uncertainty for fixed $m_c$, as seen in Fig. 9. We also note that the charm structure function at these high scales is reasonably well represented by the charm distribution, while at low scales, certainty including $Q^2=4~\mathrm{GeV}^2$, this is not true. Indeed at NNLO the boundary condition for the charm distribution is negative at very low *x* if the transition point is $m_c^2$, but this is more than compensated for by the gluon and light quark initiated cross section. As noted in [9], use of a zero mass scheme becomes unfeasible at NNLO. The dependence on the heavy-quark cross section at low scales relative to the mass is much better gauged from Fig. 7.

**Fig. 10:**
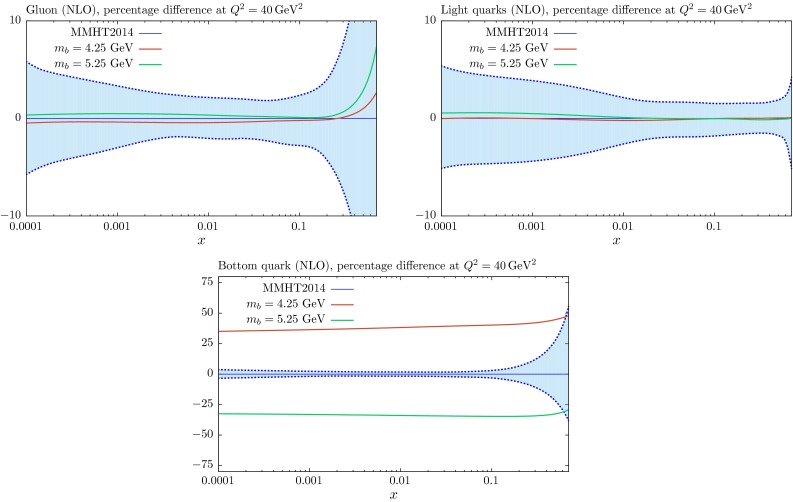
The $m_b$ dependence of the gluon, light-quark singlet and charm distributions at NLO for $Q^2=40~\mathrm{GeV}^2$, compared to the standard MMHT2014 distributions with $m_c=1.4~\mathrm{GeV}$ and $m_b=4.75~\mathrm{GeV}$

**Fig. 11:**
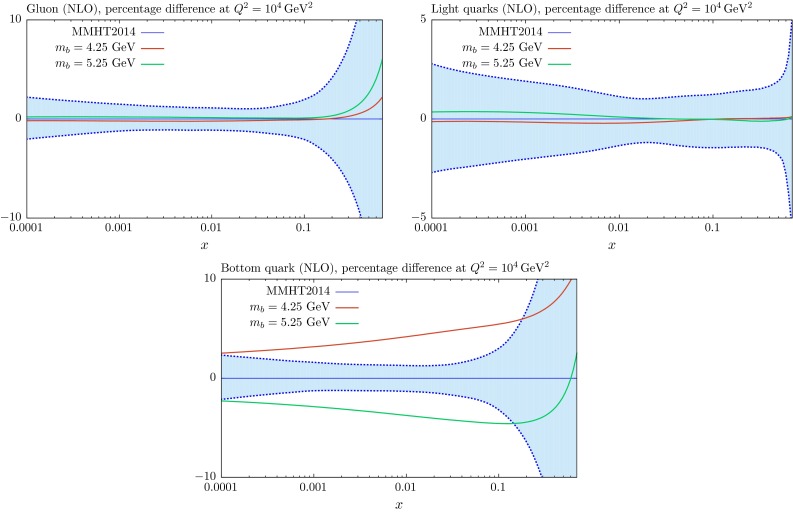
The $m_b$ dependence of the gluon, light-quark singlet and charm distributions at NLO for $Q^2=10^4~\mathrm{GeV}^2$, compared to the standard MMHT2014 distributions with $m_c=1.4~\mathrm{GeV}$ and $m_b=4.75~\mathrm{GeV}$

The relative changes in the gluon and light quarks for variations in $$m_b$$ are significantly reduced due to the much smaller impact of the beauty contribution to the structure functions from the charge-squared weighting, as can be seen in Figs. 10 and 11, where we show NLO PDFs for $$m_b=4.25$$ and $$5.25~\mathrm{GeV}$$. At $$Q^2=40\, {\mathrm{GeV}^2} \sim 2m_b^2$$ the relative change in the beauty distribution for a $$\sim $$$$ 10\,\%$$ change in the mass is similar to that for the same type of variation for $$m_c$$. However, the extent to which this remains at $$Q^2=10^4~\mathrm{GeV}^2$$ is much greater than the charm case due to the smaller evolution length.

## Effect on benchmark cross sections 

In this section we show the variation with $m_c$ and $m_b$ for cross sections at the Tevatron, and for 7 and $14~\mathrm TeV$ at the LHC. Variations for 8 and $13~\mathrm TeV$ will be very similar to those at 7 and $14~\mathrm TeV$, respectively. We calculate the cross sections for *W* and *Z* boson, Higgs boson via gluon–gluon fusion and top-quark pair production. To calculate the cross section we use the same procedure as was used in [4, 16]. That is, for *W*, *Z* and Higgs production we use the code provided by W.J. Stirling, based on the calculation in [17–19], and for top pair production we use the procedure and code of [20]. Here our primary aim is not to present definitive predictions or to compare in detail to other PDF sets, as both these results are frequently provided in the literature with very specific choices of codes, scales and parameters which may differ from those used here. Rather, our main objective is to illustrate the relative influence of varying $m_c$ and $m_b$ for these benchmark processes.

We show the predictions for the default MMHT2014 PDFs, with PDF uncertainties, and the relative changes due to changing $m_c$ from 1.25 to $1.55~\mathrm{GeV}$, and $m_b$ from 4.25 to $5.25~\mathrm{GeV}$, i.e. changing the default values by approximately $10\,\%$ in each case. The dependence of the benchmark predictions on the value of $m_c$ in Tables 3, 4 and 5 reflects the behaviour of the gluon with $\sqrt{s}$. The changes in cross section generally scale linearly in variation of masses away from the default values to a good approximation, although for $m_b$, where the cross section sensitivity to the mass choice is often small, this is less true, and in some cases the cross section is even found to decrease or increase in both directions away from the best fit mass.

We begin with the predictions for the *W* and *Z* production cross sections. The results at NNLO are shown in Table 3. The PDF uncertainties on the cross sections are $2\,\%$ at the Tevatron and slightly smaller at the LHC—the lower beam energy at the Tevatron meaning the cross sections have more contribution from higher *x* where the PDF uncertainties increase. The $m_c$ variation is at most about $0.4\,\%$ at the Tevatron and is 0.5–1$\,\%$ at the LHC, being larger at 14 TeV. The results at NLO are very similar.

In Table 4 we show the analogous results for the top-quark pair production cross section. At the Tevatron the PDFs are probed in the region $x\approx 0.4/1.96\approx 0.2$, and the main production is from the $q{\bar{q}}$ channel. At the LHC the dominant production at higher energies (and with a proton–proton rather than proton–antiproton collider) is gluon–gluon fusion, with the central *x* value probed being $x\approx 0.4/7 \approx 0.06$ at 7 TeV, and $x\approx 0.4/14\approx 0.03$ at 14 TeV. The PDF uncertainties on the cross sections are nearly $3\,\%$ at the Tevatron, similar for 7 TeV at the LHC, but a little smaller at 14 TeV as there is less sensitivity to the high-*x* gluon. The $m_c$ variation are slightly less than $1\,\%$ at the Tevatron and for 7 TeV at the LHC, but rather lower at 14 TeV since the *x* probed is near the fixed point for the gluon (see Fig. 9).

In Table 5 we show the uncertainties in the rate of Higgs boson production from gluon–gluon fusion. At the Tevatron the dominant *x* range probed, i.e. $$x\approx 0.125/1.96 \approx 0.06$$, corresponds to a region where the gluon distribution falls as $$m_c$$ increases and at the LHC where $$x \approx 0.01$$–0.02 at central rapidity the gluon increases as $$m_c$$ increases, though at 7 TeV we are only just below the fixed point. At the Tevatron the resultant uncertainty is $$\sim $$$$ 0.7\,\%$$. At the LHC at 7 TeV it is in the opposite direction but only $$\sim $$$$ 0.1\,\%$$, whereas at 14 TeV it has increased to near $$0.5\,\%$$.

As in [5] we recommend that in order to estimate the total uncertainty due to PDFs and the quark masses it is best to add the variation due to the variation in quark mass in quadrature with the PDF uncertainty, or the PDF$$+$$$$\alpha _S$$ uncertainty, if the $$\alpha _S$$ uncertainty is also used.

## PDFs in three- and four-flavour-number schemes

In our default studies we work in a general-mass variable-flavour-number scheme (GM-VFNS) with a maximum of five active flavours. This means that we start at our input scale of $Q_0^2=1~\mathrm{GeV}^2$ with three active light flavours. At the transition point $m_c^2$ the charm quark starts evolution and then at $m_b^2$ the beauty quark also starts evolution. The evolution is in terms of massless splitting functions, and at high $Q^2$ the contribution from charm and bottom quarks lose all mass dependence other than that in the boundary conditions at the chosen transition point. The explicit mass dependence is included at lower scales, but falls away like inverse powers as $Q^2/m^2_{c,b} \rightarrow \infty$. We do not currently ever consider the top quark as a parton.

We could alternatively keep the information about the heavy quarks only in the coefficient functions, i.e. the heavy quarks would only be generated in the final state. This is called a fixed-flavour-number scheme (FFNS). One example would be where neither charm and beauty exist as partons. This would be a three-flavour FFNS. An alternative would be to turn on charm evolution but never allow beauty to be treated as a parton. This is often called a four-flavour FFNS. We will use this notation, but strictly speaking it is a GM-VFNS with a maximum of four active flavours.

One might produce the partons for the three- and four-flavour FFNS by performing global fits in these schemes. However, it was argued in [21] that the fit to structure function data is not optimal in these schemes. Indeed, evidence for this has been provided in [9, 22, 23]. Moreover, much of the data (for example, on inclusive jets and *W*, *Z* production at hadron colliders) is not known to NNLO in these schemes, and is very largely at scales where $m_{c,b}$ are relatively very small. So it is clear that the GM-VFNS are more appropriate. Hence, in [24] it was decided to make available PDFs in the three- and four-flavour schemes simply by using the input PDFs obtained in the GM-VFNS, but with evolution of the beauty quark, or both the beauty and the charm quark, turned off. This procedure was continued in [5] and is the common choice for PDF groups who fit using a GM-VFNS but make PDFs available with a maximum of three or four active flavours. Hence, here, we continue to make this choice for the MMHT2014 PDFs.

We make PDFs available with a maximum of three or four active flavours for the NLO central PDFs and their uncertainty eigenvectors for both the standard choices of $\alpha ^{n_{f,\max }=5}_S(M_Z^2)$ of 0.118 and 0.120, and for the NNLO central PDF and the uncertainty eigenvectors for the standard choice of $\alpha ^{n_{f,\max }=5}_S(M_Z^2)$ of 0.118. We also provide PDF sets with $\alpha _S(M_Z^2)$ displaced by 0.001 from these default values, so as to assist with the calculation of $\alpha _s$ uncertainties in the different flavour schemes. Finally, we make available PDF sets with different values of $m_c$ and $m_b$ in the different fixed-flavour schemes.

**Table 3:** Predictions for $W^\pm$ and *Z* cross sections (in nb), including leptonic branching, obtained with the NNLO MMHT2014 parton sets. The PDF uncertainties and $m_c$ and $m_b$ variations are also shown, where the $m_c$ variation corresponds to $\pm 0.15~\mathrm{GeV}$ and the $m_b$ variation corresponds to $\pm 0.5~\mathrm{GeV}$, i.e. about $10\,\%$ in each case

	$\sigma$	PDF unc.	$m_c$ var.	$m_b$ var.
$W\,\, \mathrm{Tevatron}\,\,(1.96~\mathrm TeV)$	2.78	${}^{+0.0017}_{-0.056}$ $\left( {}^{+2.0\,\%}_{-2.0\,\%}\right)$	${}^{+0.0017}_{-0.0086}$ $\left( {}^{+0.061\,\%}_{-0.31\,\%}\right)$	${}^{-0.00092}_{-0.0015}$ $\left( {}^{-0.033\,\%}_{-0.052\,\%}\right)$
$Z \,\,\mathrm{Tevatron}\,\,(1.96~\mathrm TeV)$	0.256	${}^{+0.0052}_{-0.0046}$ $\left( {}^{+2.0\,\%}_{-1.8\,\%}\right)$	${}^{+0.00042}_{-0.0011}$ $\left( {}^{+0.16\,\%}_{-0.43\,\%}\right)$	${}^{-0.00029}_{-0.000016}$ $\left( {}^{-0.11\,\%}_{-0.0059\,\%}\right)$
$W^+ \,\,\mathrm{LHC}\,\, (7~\mathrm TeV)$	6.20	${}^{+0.103}_{-0.092}$ $\left( {}^{+1.7\,\%}_{-1.5\,\%}\right)$	${}^{+0.029}_{-0.040}$ $\left( {}^{+0.48\,\%}_{-0.64\,\%}\right)$	${}^{+0.0043}_{-0.014}$ $\left( {}^{+0.070\,\%}_{-0.22\,\%}\right)$
$W^- \,\,\mathrm{LHC}\,\, (7~\mathrm TeV)$	4.31	${}^{+0.067}_{-0.076}$ $\left( {}^{+1.6\,\%}_{-1.8\,\%}\right)$	${}^{+0.019}_{-0.022}$ $\left( {}^{+0.44\,\%}_{-0.51\,\%}\right)$	${}^{+0.0059}_{-0.0091}$ $\left( {}^{+0.14\,\%}_{-0.21\,\%}\right)$
$Z \,\,\mathrm{LHC}\,\, (7~\mathrm TeV)$	0.964	${}^{+0.014}_{-0.013}$ $\left( {}^{+1.5\,\%}_{-1.3\,\%}\right)$	${}^{+0.0074}_{-0.0088}$ $\left( {}^{+0.77\,\%}_{-0.92\,\%}\right)$	${}^{-0.00096}_{-0.00038}$ $\left( {}^{-0.10\,\%}_{-0.039\,\%}\right)$
$W^+ \,\,\mathrm{LHC}\,\, (14~\mathrm TeV)$	12.5	${}^{+0.22}_{-0.18}$ $\left( {}^{+1.8\,\%}_{-1.4\,\%}\right)$	${}^{+0.091}_{-0.12}$ $\left( {}^{+0.73\,\%}_{-0.93\,\%}\right)$	${}^{+0.0087}_{-0.037}$ $\left( {}^{+0.069\,\%}_{-0.30\,\%}\right)$
$W^- \,\,\mathrm{LHC}\,\, (14~\mathrm TeV)$	9.3	${}^{+0.15}_{-0.14}$ $\left( {}^{+1.6\,\%}_{-1.5\,\%}\right)$	${}^{+0.064}_{-0.075}$ $\left( {}^{+0.69\,\%}_{-0.81\,\%}\right)$	${}^{+0.012}_{-0.029}$ $\left( {}^{+0.13\,\%}_{-0.31\,\%}\right)$
$Z \,\,\mathrm{LHC}\,\, (14~\mathrm TeV)$	2.06	${}^{+0.035}_{-0.030}$ $\left( {}^{+1.7\,\%}_{-1.5\,\%}\right)$	${}^{+0.021}_{-0.025}$ $\left( {}^{+1.03\,\%}_{-1.2\,\%}\right)$	${}^{-0.0035}_{-0.0013}$ $\left( {}^{-0.17\,\%}_{-0.062\,\%}\right)$

**Table 4:** Predictions for $t\overline{t}$ cross sections (in nb), obtained with the NNLO MMHT2014 parton sets. The PDF uncertainties and $m_c$ and $m_b$ variations are also shown, where the $m_c$ variation corresponds to $\pm 0.15~\mathrm{GeV}$ and the $m_b$ variation corresponds to $\pm 0.5~\mathrm{GeV}$

	$\sigma$	PDF unc.	$m_c$ var.	$m_b$ var.
$t\overline{t}$ $\mathrm{Tevatron}\,\,(1.96~\mathrm TeV)$	7.5	${}^{+0.21}_{-0.20}$ $\left( {}^{+2.8\,\%}_{-2.7\,\%}\right)$	${}_{+0.077}^{-0.059}$ $\left( {}_{+1.0\,\%}^{-0.78\,\%}\right)$	${}_{+0.0015}^{+0.0088}$ $\left( {}_{+0.20\,\%}^{+0.12\,\%}\right)$
$t\overline{t}$ $\mathrm{LHC}\,\, (7~\mathrm TeV)$	176	${}^{+3.9}_{-5.5}$ $\left( {}^{+2.2\,\%}_{-3.1\,\%}\right)$	${}_{+1.4}^{-1.1}$ $\left( {}_{+0.77\,\%}^{-0.60\,\%}\right)$	${}_{-0.009}^{+0.77}$ $\left( {}_{-0.0051\,\%}^{+0.44\,\%}\right)$
$t\overline{t}$ $\mathrm{LHC}\,\, (14~\mathrm TeV)$	970	${}^{+16}_{-20}$ $\left( {}^{+1.6\,\%}_{-2.1\,\%}\right)$	${}_{+3.1}^{-3.0}$ $\left( {}_{+0.32\,\%}^{-0.31\,\%}\right)$	${}_{-1.7}^{+3.1}$ $\left( {}_{+0.17\,\%}^{-0.32\,\%}\right)$

**Table 5:** Predictions for the Higgs boson cross sections (in nb), obtained with the NNLO MMHT 2014 parton sets. The PDF uncertainties and $m_c$ and $m_b$ variations are also shown, where the $m_c$ variation corresponds to $\pm 0.15~\mathrm{GeV}$ and the $m_b$ variation corresponds to $\pm 0.5~\mathrm{GeV}$

	$\sigma$	PDF unc.	$m_c$ var.	$m_b$ var.
Higgs $\mathrm{Tevatron}\,\,(1.96~\mathrm TeV)$	0.87	${}^{+0.024}_{-0.030}$ $\left( {}^{+2.7\,\%}_{-3.4\,\%}\right)$	${}^{-0.0060}_{+0.0070}$ $\left( {}^{-0.68\,\%}_{+0.79\,\%}\right)$	${}^{+0.0042}_{-0.0011}$ $\left( {}^{+0.48\,\%}_{-0.13\,\%}\right)$
Higgs $\mathrm{LHC}\,\, (7~\mathrm TeV)$	14.6	${}^{+0.21}_{-0.29}$ $\left( {}^{+1.4\,\%}_{-2.0\,\%}\right)$	${}^{+0.025}_{-0.019}$ $\left( {}^{+0.17\,\%}_{-0.13\,\%}\right)$	${}^{+0.049}_{-0.044}$ $\left( {}^{+0.34\,\%}_{-0.30\,\%}\right)$
Higgs $\mathrm{LHC}\,\, (14~\mathrm TeV)$	47.7	${}^{+0.63}_{-0.88}$ $\left( {}^{+1.3\,\%}_{-1.8\,\%}\right)$	${}^{+0.27}_{-0.22}$ $\left( {}^{+0.57\,\%}_{-0.48\,\%}\right)$	${}^{+0.16}_{-0.16}$ $\left( {}^{+0.34\,\%}_{-0.33\,\%}\right)$

By default, when the charm or beauty quark evolution is turned off, we also turn off the contribution of the same quark to the running coupling. This is because most calculations use this convention when these quarks are entirely final state particles. This results in the coupling running more quickly. So if the coupling at $Q_0^2$ is chosen so that $\alpha ^{n_{f,\max }=5}_S(M_Z^2)\approx 0.118$, then we find that $\alpha ^{n_{f,\max }=3}_S(M_Z^2)\approx 0.105$ and $\alpha ^{n_{f,\max }=4}_S(M_Z^2)\approx 0.113$. There are sometimes cases where a set of PDFs with no beauty quark but with five-flavour running coupling is desired, e.g. [25]. After the publication of [5], PDF sets with this definition were made available. Here we make available PDFs for the central sets together with their eigenvectors with a maximum of four active flavours, but the beauty quark included in the running of the coupling. This type of PDF has also been considered very recently in [26].

**Fig. 12:**
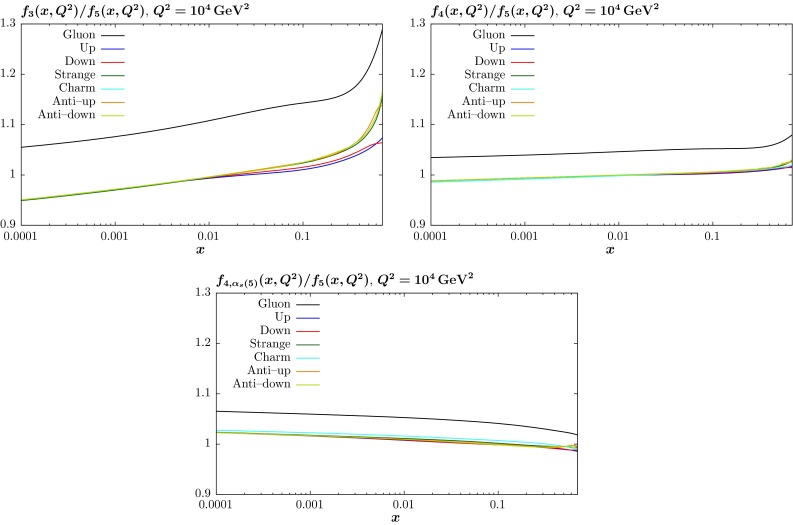
The ratio of the different fixed-flavour PDFs to the standard five flavour PDFs at NNLO and at $Q^2=10^4~\mathrm{GeV}^2$. The three and four flavour schemes are show in the *top left* and *right plots*, while the four flavour scheme with five flavours in the running of $\alpha _S$ is shown in the *bottom plot*

The variation of the PDFs defined with a maximum number of three and four flavours, compared to our default of five flavours, is shown at $Q^2=10^4~\mathrm{GeV}^2$ in Fig. 12 for NNLO PDFs. The general form of the differences are discussed in detail in section 4 of [5] and are primarily due to two effects. For fewer active quarks there is less gluon branching, so the gluon is larger if the flavour number is smaller. Also, as $Q^2$ increases the coupling gets smaller for fewer active quarks, so evolution is generally slower, which means partons decrease less quickly for large *x* and grow less quickly at small *x*. The latter effect dominates for quark evolution, while for the gluon the two effects compete at small *x*. For the case where the maximum number of flavours is 4, but the coupling has five-flavour evolution, the overwhelming effect is that the gluon is larger—effectively replacing the missing beauty quarks in the momentum sum rule. However, the increase in the gluon is maximal at small *x*, where the increased coupling compared to the case where we use the four-flavour coupling leading to increased loss of gluons at high *x* from evolution.

## Renormalisation schemes

At present most PDF fitting groups, including the most recent updates [4, 27–29], use the pole mass definition for the heavy quarks. Hence, we have remained with this definition in our investigation of quark mass dependence in this article. The analyses in [30, 31] use the $\overline{\mathrm{MS}}$ definition, following the developments in [32]. The latter analyses perform their fits in the fixed-flavour-number scheme (FFNS), while all the others groups use a general-mass variable-flavour-number scheme. There is no fundamental obstacle to switching between the two renormalisation schemes using either approach. The mass dependence in a GM-VFNS appears in entirety from the FFNS coefficient functions and in the transition matrix elements which set the boundary conditions for the (massless) evolution of the charm and beauty quarks. These are used along with the FFNS coefficient functions to define the GM-VFNS coefficient functions which tend to the massless versions as $m_{c,b}^2/Q^2 \rightarrow 0$. Under a change in the definition of the quark mass^2^

2 $$\begin{aligned} m^\mathrm{pole} = m(\mu _R)(1+\alpha _S(\mu _R^2)d^1(\mu _R^2) + \cdots ) \end{aligned}$$

the coefficient functions and transition matrix elements can be transformed from one mass scheme to the other straightforwardly, as illustrated in Eq. (8) of [32], and the mass in GM-VFNS defined in the $\overline{\mathrm{MS}}$ renormalisation scheme.

However, there is more sensitivity to the definition of the mass in a FFNS at given order than in a GM-VFNS. At LO there is no mass scheme dependence in the same way that there is no renormalisation scheme dependence of any sort. At NLO in the FFNS the variation of the LO $\mathcal{O}(\alpha _S)$ coefficient function under the change in Eq. (2) leads to a change in the NLO $\mathcal{O}(\alpha _S)$ coefficient function. Some NLO GM-VFNS definitions (e.g. the SACOT($\chi$) [33] and the FONLL-A [34]) only use the FFNS coefficient functions at $\mathcal{O}(\alpha _S)$. The transition matrix element for heavy-quark evolution in an NLO GM-VFNS is also defined at $\mathcal{O}(\alpha _S)$ (and indeed is zero with the standard choice $\mu _F=m_{c,b}$), so neither depend on the mass definition, and the NLO GM-VFNS is independent of the mass scheme [35].

Some NLO GM-VFNS definitions do use the $\mathcal{O}(\alpha _S^2)$ FFNS coefficient functions. Hence, these will contain some dependence on the mass scheme. However, in the original TR [36] and then the TR’ [10] schemes this contribution is frozen at $Q^2=m_{c,b}^2$, so becomes relatively very small at high $Q^2$. In the “optimal” TR’ scheme [9], and in the FONLL-B, the dependence falls away like $m_{c,b}^2/Q^2$ (in the former case the whole $\mathcal{O}(\alpha _S^2)$ coefficient function is weighted by $m_{c,b}^2/Q^2$, while in the FONLL-B scheme the subtraction means that only the massless limit of the $\mathcal{O}(\alpha _S^2)$ coefficient function remains as $m_{c,b}^2/Q^2\rightarrow 0$). Hence, the dependence on the mass scheme is more limited than in the FFNS at NLO, and is particularly small. Indeed, in all but the original TR and TR’ schemes, there is no dependence at high $Q^2$.

At NNLO the mass scheme dependence in the FFNS enters in the $\mathcal{O}(\alpha _S^2)$ and $\mathcal{O}(\alpha _S^3)$ coefficient functions. In a GM-VFNS it now enters in the $\mathcal{O}(\alpha _S^2)$ coefficient functions at low scales, and in boundary conditions for evolution, which gives effects which persist to all scales. If the GM-VFNS uses the $\mathcal{O}(\alpha _S^3)$ coefficient functions these will also give mass scheme dependent effects at low $Q^2$. However, the expressions for the $\mathcal{O}(\alpha _S^3)$ coefficient functions are themselves still approximations [37].

Hence, at present it does not seem too important whether the pole mass or $\overline{\mathrm{MS}}$ renormalisation scheme is used in a GM-VFNS (indeed in [27] the pole mass scheme is used, but the $\overline{\mathrm{MS}}$ values for the masses are taken). Nevertheless, in the future it is probably ideal to settle on the $\overline{\mathrm{MS}}$ mass, since the value of this is quite precisely determined in many experiments, which is not true of the pole mass. At the same time it will also be desirable for different PDF groups to agree on a common value of $m_c$ and $m_b$ (there is no agreement at present).

## Conclusions

The main purpose of this article is to present and make available PDF sets in the framework used to produce the MMHT2014 PDFs, but with differing values of the charm and beauty quark mass. We do not make a determination of the optimum values of these masses, but we do investigate and note the effect the mass variation has on the quality of the fits to the data, concentrating on the HERA cross section data with charm or beauty in the final state. We note that for both the charm and beauty quarks a lower mass than our default values of $m_c=1.4~\mathrm{GeV}$ and $m_b=4.75~\mathrm{GeV}$ is preferred, although these are roughly the values of pole masses one would expect from conversion from the values measured in the $\overline{\mathrm{MS}}$ scheme. This suggests that in the future it may be better to use the $\overline{\mathrm{MS}}$ definition, though this is currently not the practice in global fits using a GM-VFNS—perhaps because, as we discuss, the mass scheme dependence has less effect in these schemes than for the FFNS. We also make PDFs available with a maximum of three or four active quark flavours. The PDF sets obtained for different quark masses and for different active quark flavours can be found at [38] and will be available from the LHAPDF library [39].

We investigate the variation of the PDFs and the predicted cross sections for standard processes at the LHC (and Tevatron) corresponding to these variations in heavy-quark mass. For reasonable variations of $m_c$ the effects are small, but not insignificant, compared to PDF uncertainties. For variations in $m_b$ the effect is smaller, and largely insignificant, except for the beauty distribution itself, which can vary more than its uncertainty at a fixed value of $m_b$; see, in particular Fig. 10. Hence, currently the uncertainties on PDFs due to quark masses are not hugely important, but need to be improved in the future for very high precision predictions at hadron colliders.

## Footnotes

1 As discussed in [1], for a $$68\,\%$$ confidence level uncertainty we insist the fit quality to a given data set deteriorates by no more than the width of the $$\chi ^2$$ distribution for *N* points, roughly $$\sqrt{N/2}$$ multiplied by the $$\chi ^2$$ per point for the best fit. For the charm cross section data this is $$\approx $$$$ \sqrt{52/2}\times 1.3$$.

2 Note that $d^1(\mu _R^2)=4/3\pi$ if $\mu _R=m$.
